# Erratum to: Morphometric analysis of *Passiflora* leaves: the relationship between landmarks of the vasculature and elliptical Fourier descriptors of the blade

**DOI:** 10.1093/gigascience/gix070

**Published:** 2017-10-06

**Authors:** Daniel H. Chitwood, Wagner C. Otoni

**Keywords:** *Passiflora*, Morphometrics, Leaves, Leaf shape, Leaf morphology, Heteroblasty, Procrustes analysis, Landmarks, Elliptical Fourier Descriptors

## Abstract

**Background:**

Leaf shape among *Passiflora* species is spectacularly diverse. Underlying this diversity in leaf shape are profound changes in the patterning of the primary vasculature and laminar outgrowth. Each of these aspects of leaf morphology—vasculature and blade—provides different insights into leaf patterning.

**Results:**

Here, we morphometrically analyze >3300 leaves from 40 different *Passiflora* species collected sequentially across the vine. Each leaf is measured in two different ways: using 1) 15 homologous Procrustes-adjusted landmarks of the vasculature, sinuses, and lobes; and 2) Elliptical Fourier Descriptors (EFDs), which quantify the outline of the leaf. The ability of landmarks, EFDs, and both datasets together are compared to determine their relative ability to predict species and node position within the vine. Pairwise correlation of x and y landmark coordinates and EFD harmonic coefficients reveals close associations between traits and insights into the relationship between vasculature and blade patterning.

**Conclusions:**

Landmarks, more reflective of the vasculature, and EFDs, more reflective of the blade contour, describe both similar and distinct features of leaf morphology. Landmarks and EFDs vary in ability to predict species identity and node position in the vine and exhibit a correlational structure (both within landmark or EFD traits and between the two data types) revealing constraints between vascular and blade patterning underlying natural variation in leaf morphology among *Passiflora* species.

**Correction**

In the final publication of “Morphometric analysis of *Passiflora* leaves: the relationship between landmarks of the vasculature and elliptical Fourier descriptors of the blade,” by Daniel H. Chitwood and Wagner C. Otoni, the listed accepted date was incorrect, and the article did not include the revised date. The accepted date has been corrected and the revised date added. The corrected manuscript can be found online at https://academic.oup.com/gigascience/article/6/1/1/2865207/Morphometric-analysis-of-Passiflora-leaves-the?searchresult=1.

The Publisher regrets these errors.

