# Supplementary material for: Morphometric analysis of Passiflora leaves: the relationship between landmarks of the vasculature and elliptical Fourier descriptors of the blade
Source: Gigascience. 2017 Jan 7;6(1):1–13. doi: 10.1093/gigascience/giw008 (PMC5437945; doi:10.1093/gigascience/giw008)
Supplement: Reviewer_2_Original_Submission_(Attachment).pdf [file giw008_Reviewer_2_Original_Submission_(Attachment).pdf]

Review of GIGA-D-16-00069, "Morphometric analysis of *Passiflora* leaves I: the relationship between landmarks of the vasculature and elliptical Fourier descriptors of the blade" by D.H. Chitwood and W.C. Otoni.

This is a valuable contribution, both in terms of (a) the data collected (leaf outlines and landmarks from a large sample of *Passiflora* species exhibiting a wide range of leaf shapes), and (b) the well thought out analyses undertaken with care and, for the most part, attention to detail. The paper also showcases, at least for me, the range of tools now available for comparative biologists and others who seek to analyze patterns of morphological variation and ontogenetic change. Until relatively recently it appeared that there was no real replacement for MorphoSys (Meacham, 1993) and the ability of this MS-DOS based system for outline capture and measurement. In many ways there still isn't, leaving workers to pick through an assortment of programs with more limited scopes or (better), to learn to use the imageJ toolbox effectively (Schneider, Rasband, and Eliceiri, 2012). Or SHAPE (Iwata and Ukai, 2002), if only outline data are needed. And in the meantime, adepts from the morphometrics world have embraced R (R_Core_Team, 2016) as a data analysis and graphics environment, resulting in packages like *shapes* (Dryden, 2016) and *Momocs* (Bonhomme et al., 2014).

Others (e.g. Jensen, Ciofani, and Miramontes, 2002), myself included, have compared results from different analytical methods based, variously, on landmark and outline data from a common sample of study objects. The authors' correlation analysis (Fig. 7) is especially valuable (and as far as I know entirely original) for the way it enables the authors to infer connections between particular landmarks (their *x* or *y* coordinates) and particular elliptic Fourier coefficients (A, B, C, or D) for a given harmonic. Although they tend to contrast their landmark data as reflecting leaf vasculature features (because these provide the locations of several of their landmarks), as against overall shape features provided by their outline data, the authors also emphasize the complementarity of these two aspects of their study objects (p. 5). In their subsequent ms (GIGA-D-16-00070) the authors are more adroit in emphasizing this complementarity. Here, they occasionally sound as if they think these associations are somehow intrinsic in these data sources, as if they would obtain even in objects other than leaves (pp. 2, 3, 6). It may be that what's at issue here is just unnecessarily bringing forward *interpretations* of their results (as if they described the results) before those results have been fully presented.

I have three concerns about this ms. First, the authors have been variously careless about details of spelling (“fratal,” “heteroblatsy”) and the agreement between verb and noun (“data” is a plural noun) and somewhat cavalier about explaining terms when first introduced (“eigenleaf”; “amplification factor,” in the caption to Fig. 1).

Second, basic details of the authors’ sampling are unclear, probably because they are so abundantly obvious to them. What is the actual sample size for their study? They refer (pp. 2, 4, 5, 10) to having analyzed more than 3,300 leaves, and (on p. 5) to making available a dataset comprising “…555 scans of leaves from 40 different species of *Passiflora*…” Presumably, the data released represent a subset of the total sample, but it would help to make this, and their overall sampling strategy, explicit by tabulating how many vines (with what numbers of leaves) were studied for each of the 40 species. These details are important in order to dispel any idea that their discriminant analyses are overfitted because of the large numbers of descriptors (30, 80, or 110) and binary-valued dummy variables for species and leaf position (39, 10); Gittins’ monograph on canonical analysis (Gittins, 1985) references simulation studies suggesting that upwards of 20 times as many study objects as descriptors (measured, plus those designating groups) are needed in order for an analysis to be anything other than a deterministic description of a particular dataset. One of the exciting aspects of this study is the refreshingly large sample size that appears to have been used.

The third, related, concern is that the way in which leaf position on individual vines was recorded is unclear, given that nothing seems to be said about whether the same number of leaves was produced on all vines of all species during the period during which the study material was grown. Numbering leaves from the youngest leaf at the tip of the shoot, to the base, suggests that the youngest leaf would be numbered 1, the next 2, and so on to *N*, the most basal leaf on the vine. If the total number of leaves varies from vine to vine then there won’t be an exact homology between leaf positions. Figures 5 and 6 suggest that leaf position 1 (basal-most, left-most, respectively) is in fact the most basal leaf. Would this position in fact correspond to the cotyledons, or to the first post-cotyledonary leaf? Clarification would be helpful. I also wonder about the continued reference to “node position” and “heteroblastic node position” as well as, in one case, “shoot position.” I suggest that a more transparent usage would be to refer throughout to leaf position (numbered from the most basal leaf), or to the position of a leaf on a shoot. Reference to “heteroblastic node position” seems completely unnecessary, since heteroblasty is an

emergent property of some or all of the shoots studied, as seen from the way in which leaves vary over the course of shoot development in shape (or some other property) from the most basal to the most apical.

BONHOMME, V., S. PICQ, C. GAUCHEREL & J. CLAUDE. 2014. Momocs: Outline Analysis Using R. *Journal of Statistical Software* 56: 1-24.

DRYDEN, I. L. 2016. shapes package, version 1.1-13. website: https://www.maths.nottingham.ac.uk/personal/ild/shapes/index.html.

GITTINS, R. 1985. Canonical Analysis. Springer-Verlag, Berlin.

IWATA, H. & Y. UKAI. 2002. SHAPE: A computer program package for quantitative evaluation of biological shapes based on elliptic Fourier descriptors. *Journal of Heredity* 93: 384-385.

JENSEN, R. J., K. M. CIOFANI & L. C. MIRAMONTES. 2002. Lines, outlines, and landmarks: morphometric analyses of leaves of Acer rubrum, Acer saccharinum (Aceraceae) and their hybrid. *Taxon* 51: 475-492.

MEACHAM, C. A. 1993. *MorphoSys* An interactive machine vision program for acquisition of morphometric data. *In* R. Fortuner [ed.], *Advances in computer methods for systematic biology: Artificial intelligence, databases, computer vision*, 393-402.

R CORE TEAM. 2016. R: A language and environment for statistical computing. website: http://www.R-project.org.

SCHNEIDER, C. A., W. S. RASBAND & K. W. ELICEIRI. 2012. NIH Image to ImageJ: 25 years of image analysis. *Nature Methods* 9: 671-675.
