# Supplementary material for: Morphometric analysis of Passiflora leaves: the relationship between landmarks of the vasculature and elliptical Fourier descriptors of the blade
Source: Gigascience. 2017 Jan 7;6(1):1–13. doi: 10.1093/gigascience/giw008 (PMC5437945; doi:10.1093/gigascience/giw008)
Supplement: Reviewer_2_Revision_1(_Attachment).pdf [file giw008_Reviewer_2_Revision_1(_Attachment).pdf]

Review of “Morphometric analysis of Passiflora leaves: the relationship between landmarks of the vasculature and elliptical Fourier descriptors of the blade” (revision 1), by D. H. Chitwood and W. C. Otoni.

The authors have responded to my comments, and for the most part have satisfied my concerns. In this regard, the copy of the original ms marked up to show the changes the authors have made is extremely helpful. The material on github consists of the data files and R code used to produce the figures, and does not appear provide direct access to the original leaf spectra that would show the variation observed in outline and landmark position. For the publication, I trust that the journal will require that the raw data referenced as “44. Giga DB reference” will consist of a series of files, e.g. one for each species, comprising complete leaf spectra, with the leaves labeled acropetally, i.e. in the heteroblastic sequence from base to tip, for each plant referenced in Figure S1A. If these are provided as vector files (outlines plus landmarks) this data repository should not be excessively large. The vector format will also make it possible for the interested reader to zoom in and out without loss of resolution in order to examine the patterns of variation at different scales.

In what follows, the page numbers referred to are those on the marked-up copy of the original that shows the changes made in the revision.

p. 2, the authors state that “profound changes in the patterning of the primary vasculature and laminar outgrowth” underlie the diversity of leaf shape in *Passiflora*. How is it *background* knowledge that this diversity depends on profound changes in “the patterning of the primary vasculature and laminar outgrowth”? Maybe only small changes in the timing, extent, or directionality of cell division and vascular differentiation are needed to effect profound differences in shape either sequentially along a shoot or between species. It might be more appropriate to suggest that the authors’ morphometric analyses may help enable discovery of the processes underlying the diversity of leaf shapes seen within individual shoots on the one hand, and between species on the other.

p. 3, The authors write that within and between species variation in leaf shape reflects “…both the heteroblastic development of the shoot apical meristem from which they are derived and the ontogeny of individual leaves as they allometrically expand [7-10].” Earlier, in my review of the authors’ companion ms, GIGA-D-16-00070, I commented on the authors’ repeated references to heteroblasty as

resulting "...from the temporal development of the shoot apical meristem, …" and asked why they emphasized the shoot apical meristem, when (I believe) there is abundant evidence for processes determining shape and venation operating in the developing leaf primordial. Heteroblasty may or may not also reflect progressive changes in the organization of the shoot apical meristem, but whatever the case, a somewhat less facile discussion would be welcome in both mss.

p. 4 (and pp. 12, 13), The authors refer to their analytical results revealing (developmental) constraints on leaf shape and vascular pattern, referring to Fig. 7 and 8. My general reaction to this is to observe that they have achieved a sophisticated ***description*** of leaf shape variation in *Passiflora*, but in my opinion are overly optimistic in suggesting that their results demonstrate process-level constraints. Nevertheless, their example of the close relationships between y-components of landmark 11 and the x-components of landmarks 9 and 13 would support their assertion even better if Fig. 8 could somehow incorporate the sign differences hidden in Fig. 7. It struck me that if lengthwise (y-axis) expansion of the median lobe could be shown to be associated with contraction of the distal lobes along the x-axis then the authors could well speak of a constraint (conservation of leaf surface area). Such inverse relationships are hard to see, as they require examination of fine details of Fig. 7, with no help from the vectors in Fig. 8 (no indication whether the correlations coded by the orange color are positive or negative).

p. 11, In the Iwata et al. paper (the authors' reference 31) it appeared to me that the observation that the A and D coefficients of the harmonics related to asymmetry was an empirical one. Is there an analytical reason why this should be so? Did the authors carry out the same kind of separate analyses of their EFA data (comparisons of PCAs of the A and D coefficients, and of the B and C coefficients)?

pp. 5, 14, The description of the numbering system used ("The numbers written near each leaf…" on p. 14) suggests the reader will have access to the actual scans made by the authors)as their referemnce 45?). Is this in fact the case, and is it necessary? I've suggested above that the authors instead provide vector illustrations of their data (outlines and landmarks), numbered from base to apex of the shoot. The authjors need never confuse their readers with references to how leaves were initially numbered (i.e. in the opposite direction) since that numbering bears no relationship to the heteroblasty that is the subject of the authors' GigaScience mss.
