# Supplementary figures and images for: Morphometric analysis of Passiflora leaves: the relationship between landmarks of the vasculature and elliptical Fourier descriptors of the blade
Source: Gigascience. 2017 Jan 7;6(1):1–13. doi: 10.1093/gigascience/giw008 (PMC5437945; doi:10.1093/gigascience/giw008)

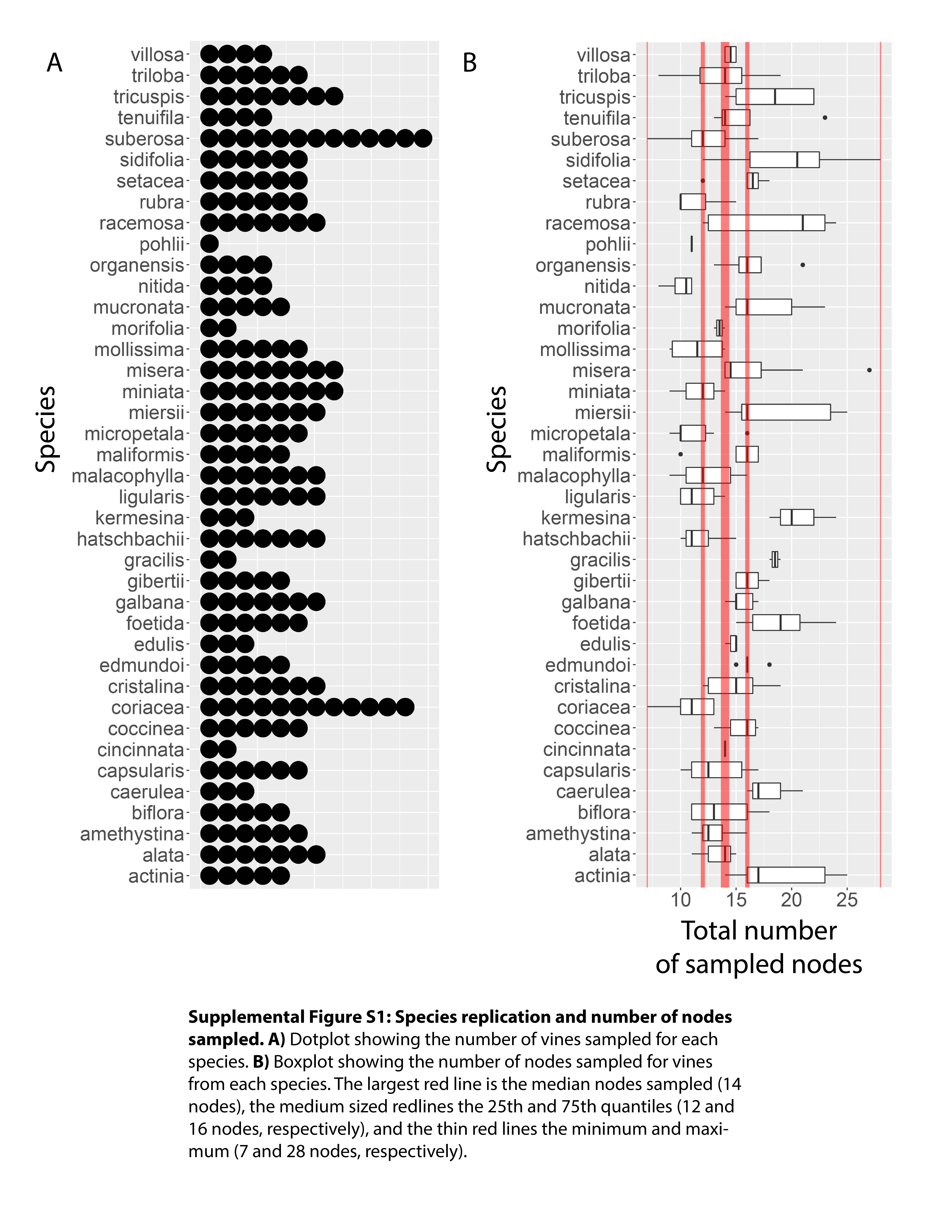

Supplement: Supplemental material — Additional file 1: Fig. S1 Species replication and number of nodes sampled. A) Dotplot showing the number of vines sampled for each species. B) Boxplot showing the number of nodes sampled for vines from each species. The largest red line is the median nodes sampled (14 nodes), the medium sized redlines the 25th and 75th quantiles (12 and 16 nodes, respectively), and the thin red lines the minimum and maximum (7 and 28 nodes, respectively). (JPG 4308 kb) [file giw008_Supp.jpeg]
