# Supplementary material for: Morphometric analysis of Passiflora leaves: the relationship between landmarks of the vasculature and elliptical Fourier descriptors of the blade
Source: Gigascience. 2017 Jan 7;6(1):1–13. doi: 10.1093/gigascience/giw008 (PMC5437945; doi:10.1093/gigascience/giw008)
Supplement: Response_to_Reviewer_Comments_Original_Submission.pdf [file giw008_Response_to_Reviewer_Comments_Original_Submission.pdf]

Reviewer #1: This paper assesses leaf shape variation in Passiflora using two different morphometric methods. I will say from the start that I am not an expert in these techniques and am not in a position to review the methods or statistics in this paper, so my comments are somewhat superficial and the paper should also be reviewed by a morphometrics/statistical expert.

--Response:
Thank you for your insightful comments! They have improved the manuscript!

Background: I'd like to see a bit more justification for the study- Is this aimed at developing automated methods for species identification, or quantifying ecological communities and their diversity of leaf shape? Or is it purely an assessment of the methods for shape quantification (Passiflora have such dramatic shape variation it should be an easy test case?)

--Response:
The scope of this manuscript is purely morphometric: its aim is, using a sampling of leaves from Passiflora species collected across the vine, to compare different morphometric methods by the factors of the species and vine positions sampled. There is a rich history of using leaf morphometric data in a wide variety of disciplines, including paleobiology and paleoclimate studies, ecology, evolution, genetics, and development. We have added a short paragraph highlighting a few of these seminal works in morphometrics so that others can apply some of the topics explored in this manuscript to different fields.

The analysis considers 'species' and developmental stage 'heteroblasty' separately and as independent factors. However I think this is the wrong approach because the plasticity of leaves is highly species dependent - so in analysing plasticity, species should be included in the model, such that the interaction of stage and species is considered. It is not really surprising that when considered independently, leaf position is not a very good predictor of leaf shape because the developmental process is different in each species. Or alternatively the leaf position analysis could be conducted on each species separately, if the data are sufficient.

It is interesting that more juvenile leaves show less species-specificity as detected in the dataset - anecdotally I have noticed the same thing.

--Response:
These are insightful comments, thank you. We did indeed perform linear modeling to determine species, node, and interaction effects. The results were that the heteroblastic effect was very weakly supported compared to species and interaction terms. We may include the results from this analysis in a subsequent manuscript regarding heteroblasty. However, the above linear modeling was performed for each morphometric variable alone: landmarks and harmonic coefficients. The Linear Discriminant Analysis (LDA) performed in this manuscript is a slightly different question: using all available morphometric traits, what is the ability to discriminant species without regard to vine position and vice versa? Another way of phrasing this is what linear combination of features in the leaf (sampling from all the features, not just a single one) allows us to distinguish genetic vs. developmental effects on leaf shape? That to some degree these are separate effects has important implications based on what we know about the developmental biology of leaves: different genetic pathways cause shape differences among all leaves between species versus conserved pathways responsible for heteroblastic development that cause changes in shape across the leaf series in different species with disparate leaf shapes. Going back to the point about the interaction effect: this is the main point of the subsequent manuscript we are preparing that is solely about heteroblasty. The main way that the interaction effect arises is that juvenile leaves are more similar between species, which we demonstrate quantitatively by performing a species LDA on each node position independently, just as the reviewer suggests. But again, this analysis is in the other manuscript. We have added text to the manuscript summarizing the above discussion.

Discussion: Are there any ecological implications of this work? Are the species sympatric, or where do they come from. I'd be interested to know about disparity in leaf shape among sympatric species, and whether

this might be greater than expected by chance, as would perhaps be predicted by the Heliconius coevolution hypothesis. It would be interesting to think further about which ecological questions could be addressed with these methods

--Response:
These are great questions to pursue. Unfortunately, it is out of the domain of both this manuscript and the authors' expertise. We hope that by making all data and code available, that those more versed in specific questions about Passiflora evolution and biogeography and Heliconius butterfly evolution might be able to tackle these interesting questions!

Page 3 Line 21 Refer to recent paper on shape learning
Dell'aglio, D. D., Losada, M. E. & Jiggins, C. D. Butterfly Learning and the Diversification of Plant Leaf Shape. Front. Ecol. Evol. 4, 81 (2016).

--Response:
This is an excellent paper that we missed because it is so recent. We have added the reference (to the section about different fields that morphometric analyses can be applied in the revised manuscript). Thanks!

Reviewer #2: Please see attached file for formatted version of review.

Review of GIGA-D-16-00069, "Morphometric analysis of Passiflora leaves I: the relationship between landmarks of the vasculature and elliptical Fourier descriptors of the blade" by D.H. Chitwood and W.C. Otoni.

This is a valuable contribution, both in terms of (a) the data collected (leaf outlines and landmarks from a large sample of Passiflora species exhibiting a wide range of leaf shapes), and (b) the well thought out analyses undertaken with care and, for the most part, attention to detail. The paper also showcases, at least for me, the range of tools now available for comparative biologists and others who seek to analyze patterns of morphological variation and ontogenetic change. Until relatively recently it appeared that there was no real replacement for MorphoSys (Meacham, 1993) and the ability of this MS-DOS based system for outline capture and measurement. In many ways there still isn't, leaving workers to pick through an assortment of programs with more limited scopes or (better), to learn to use the imageJ toolbox effectively (Schneider, Rasband, and Eliceiri, 2012). Or SHAPE (Iwata and Ukai, 2002), if only outline data are needed. And in the meantime, adepts from the morphometrics world have embraced R (R Core Team, 2016) as a data analysis and graphics environment, resulting in packages like shapes (Dryden, 2016) and Momocs (Bonhomme et al., 2014).

--Response:
Thanks! This was one of the main objectives of this manuscript: to compare different morphometric methods and to provide the code and raw data for others to experiment with and innovate new ways of thinking about leaf shape.

Others (e.g. Jensen, Ciofani, and Miramontes, 2002), myself included, have compared results from different analytical methods based, variously, on landmark and outline data from a common sample of study objects. The authors' correlation analysis (Fig. 7) is especially valuable (and as far as I know entirely original) for the way it enables the authors to infer connections between particular landmarks (their x or y coordinates) and particular elliptic Fourier coefficients (A, B, C, or D) for a given harmonic. Although they tend to contrast their landmark data as reflecting leaf vasculature features (because these provide the locations of several of their landmarks), as against overall shape features provided by their outline data, the authors also emphasize the complementarity of these two aspects of their study objects (p. 5). In their subsequent ms (GIGA-D-16-00070) the authors are more adroit in emphasizing this complementarity. Here, they occasionally sound as if they think these associations are somehow intrinsic in these data sources, as if they would obtain even in objects

other than leaves (pp. 2, 3, 6). It may be that what's at issue here is just unnecessarily bringing forward interpretations of their results (as if they described the results) before those results have been fully presented.

I have three concerns about this ms. First, the authors have been variously careless about details of spelling ("fratal," "heteroblatsy") and the agreement between verb and noun ("data" is a plural noun) and somewhat cavalier about explaining terms when first introduced ("eigenleaf"; "amplification factor," in the caption to Fig. 1).

--Response:
Thank you for catching these typos. We have tried to fix any mistakes that we found during re-reading and amending the manuscript. We have explained the terms “eigenleaf” and “amplification factor” upon their first usage. For the use of “data”, many have debated between the original plural noun usage and the modern usage, which includes the original but also singular and singular mass usages.

Second, basic details of the authors' sampling are unclear, probably because they are so abundantly obvious to them. What is the actual sample size for their study? They refer (pp. 2, 4, 5, 10) to having analyzed more than 3,300 leaves, and (on p. 5) to making available a dataset comprising "...555 scans of leaves from 40 different species of Passiflora..." Presumably, the data released represent a subset of the total sample, but it would help to make this, and their overall sampling strategy, explicit by tabulating how many vines (with what numbers of leaves) were studied for each of the 40 species.

--Response:
This is a very important point. This data was always available in the raw data and code posted on GitHub, but we have visualized the number of vines per species and the number of nodes sampled per vine in supplemental figure S1. There are large discrepancies in replicates per species and the nodes sampled per vine (which we make very clear in the text multiple times), which we try to address with the way we performed the linear discriminant analyses, described below:

These details are important in order to dispel any idea that their discriminant analyses are overfitted because of the large numbers of descriptors (30, 80, or 110) and binary-valued dummy variables for species and leaf position (39, 10); Gittins' monograph on canonical analysis (Gittins, 1985) references simulation studies suggesting that upwards of 20 times as many study objects
as descriptors (measured, plus those designating groups) are needed in order for an analysis to be anything other than a deterministic description of a particular dataset. One of the exciting aspects of this study is the refreshingly large sample size that appears to have been used.

--Response:
We agree with the author that we do have a relatively large sample size, especially considering that we perform LDAs on species without regard to node position (i.e., every vine is represented by multiple leaves) and on node position without regard to species (every node is represented by leaves from many vines). The point about unequal representation very much concerned us as well. This is why when predicting species or node position, a “leave-one-out” method of cross-validation was performed: that is, the LDA was performed for each leaf, leaving out the leaf in question, calculating a discriminant space, and predicting the identity of the leaf using the discriminant space from which it is excluded. This is a robust way to bypass concerns about unequal and small sample sizes. This method was present in the code posted on GitHub, but we have made abundantly clear in the text that this method of cross-validation was used.

The third, related, concern is that the way in which leaf position on individual vines was recorded is unclear, given that nothing seems to be said about whether the same number of leaves was produced on all vines of all species during the period during which the study material was grown. Numbering leaves from the youngest leaf at the tip of the shoot, to the base, suggests that the youngest leaf would be numbered 1, the next 2, and so on to N, the most basal leaf on the vine. If the total number of leaves varies from vine to vine then there won't be an exact homology between leaf positions. Figures 5 and 6 suggest that leaf

position 1 (basal-most, left-most, respectively) is in fact the most basal leaf. Would this position in fact correspond to the cotyledons, or to the first post-cotyledonary leaf? Clarification would be helpful. I also wonder about the continued reference to "node position" and "heteroblastic node position" as well as, in one case, "shoot position." I suggest that a more transparent usage would be to refer throughout to leaf position (numbered from the most basal leaf), or to the position of a leaf on a shoot. Reference to "heteroblastic node position" seems completely unnecessary, since heteroblasty is an emergent property of some or all of the shoots studied, as seen from the way in which leaves vary over the course of shoot development in shape (or some other property) from the most basal to the most apical.

--Response:
We are very sorry on this point for not providing more clarification. An important attribute of this manuscript is making all the raw, high resolution scans used publically available. In the scans, leaves are numbered “1” at the growing tip and successively upward towards the base. Because the use of the raw scans is so important, we mentioned this in the Data Description and Methods. In the manuscript, the numbering is opposite: it starts at the base and proceeds towards the tip. The reason for this is just as the reviewer correctly mentions: we wish the nodes to be numbered in alignment with the heteroblastic series, not only because that is the principal phenomenon we are interested in, but only this numbering scheme leads to nodes that are homologously related, even between vines of unequal lengths. We have explained our usage of “heteroblastic node position” in the main text, even going so far as to inform the reader that “node position is referred to as ‘heteroblasty’ as shorthand indicating that numbering of nodes begins at the shoot base”. This point is important to periodically remind the reader, and is actually more confusing if we fail to do so, as the opposite numbering scheme is more sensitive to the allometric expansion of leaves at the growing tip. The above discussion is mentioned in the Data Description, the main text, and the Methods, as well as notes of the numbering scheme placed in the relevant figure legends, and we hope that this provides clarity we failed to provide earlier.

BONHOMME, V., S. PICQ, C. GAUCHEREL & J. CLAUDE. 2014. Momocs: Outline Analysis Using R. Journal of Statistical Software 56: 1-24.
DRYDEN, I. L. 2016. shapes package, version 1.1-13. website: https://www.maths.nottingham.ac.uk/personal/ild/shapes/index.html.
GITTINS, R. 1985. Canonical Analysis. Springer-Verlag, Berlin.
IWATA, H. & Y. UKAI. 2002. SHAPE: A computer program package for quantitative evaluation of biological shapes based on elliptic Fourier descriptors. Journal of Heredity 93: 384-385.
JENSEN, R. J., K. M. CIOFANI & L. C. MIRAMONTES. 2002. Lines, outlines, and landmarks: morphometric analyses of leaves of Acer rubrum, Acer saccharinum (Aceraceae) and their hybrid. Taxon 51: 475-492.
MEACHAM, C. A. 1993. MorphoSys An interactive machine vision program for acquisition of morphometric data. In R. Fortuner [ed.], Advances in computer methods for systematic biology: Artificial intelligence, databases, computer vision, 393-402.
R CORE TEAM. 2016. R: A language and environment for statistical computing. website: http://www.R-project.org.
SCHNEIDER, C. A., W. S. RASBAND & K. W. ELICEIRI. 2012. NIH Image to ImageJ: 25 years of image analysis. Nature Methods 9: 671-675.
