# Supplementary material for: Morphometric analysis of Passiflora leaves: the relationship between landmarks of the vasculature and elliptical Fourier descriptors of the blade
Source: Gigascience. 2017 Jan 7;6(1):1–13. doi: 10.1093/gigascience/giw008 (PMC5437945; doi:10.1093/gigascience/giw008)
Supplement: Response_to_Reviewer_Comments_Revision_1.pdf [file giw008_Response_to_Reviewer_Comments_Revision_1.pdf]

Reviewer reports:
Reviewer #2: Please see formatted version of my review (attached).
Review of "Morphometric analysis of Passiflora leaves: the relationship between landmarks of the vasculature and elliptical Fourier descriptors of the blade" (revision 1), by D. H. Chitwood and W. C. Otoni.

The authors have responded to my comments, and for the most part have satisfied my concerns. In this regard, the copy of the original ms marked up to show the changes the authors have made is extremely helpful. The material on github consists of the data files and R code used to produce the figures, and does not appear provide direct access to the original leaf spectra that would show the variation observed in outline and landmark position.

Response: Thank you for your comments. The original leaf spectra and landmarks were always present in the GitHub repository. We have added the explicit path for each in the “Data Description” so that readers can find this information in the repository.

For the publication, I trust that the journal will require that the raw data referenced as "44. Giga DB reference" will consist of a series of files, e.g. one for each species, comprising complete leaf spectra, with the leaves labeled acropetally, i.e. in the heteroblastic sequence from base to tip, for each plant referenced in Figure S1A. If these are provided as vector files (outlines plus landmarks) this data repository should not be excessively large. The vector format will also make it possible for the interested reader to zoom in and out without loss of resolution in order to examine the patterns of variation at different scales.

Response: As mentioned in the above response, the complete leaf spectra and landmarks are provided on GitHub. In addition, we have uploaded the original binary outlines of each isolated individual leaf that was used to calculate EFDs. These are not provided as vector files because vector files were not used in the analysis of this manuscript nor are they necessary for subsequent analyses. The original scans will provide readers the ability to recalculate landmarks if they wish, and the isolated binary outlines will allow readers to recalculate the EFDs if they wish. Additionally, the actual landmarks and EFDs used in analysis are provided on GitHub, as well as the code to analyze them. Throughout the GitHub repository, both acropetal and basipetal numbering are provided in the data files, for the reader to choose which numbering system they prefer.

In what follows, the page numbers referred to are those on the marked-up copy of the original that shows the changes made in the revision.
p. 2, the authors state that "profound changes in the patterning of the primary vasculature and laminar outgrowth" underlie the diversity of leaf shape in Passiflora. How is it background knowledge that this diversity depends on profound changes in "the patterning of the primary vasculature and laminar outgrowth"? Maybe only small changes in the timing, extent, or directionality of cell division and vascular differentiation are needed to effect profound differences in shape either sequentially along a shoot or between species. It might be more appropriate to suggest that the authors' morphometric analyses may help enable discovery of the processes underlying the diversity of leaf shapes seen within individual shoots on the one hand, and between species on the other.

Response: Thank you for your comments. It is well established that “timing, extent, or directionality of cell division and vascular differentiation” underlie natural variation in leaf shape, although as described below, the specific associations that give rise to particular shapes are almost unknown. However, this manuscript cannot address those points. Such a detailed histological and molecular analysis falls outside the scope of this manuscript. This manuscript is inherently about morphometrics of leaves, and the comparison of two different methods that compare different morphological attributes of leaves. Therefore, I believe the presentation of natural variation within Passiflora as arising from patterning of primary vasculature and blade is accurate. It is not false that most the shape variation arises from 1) primary vascular patterning (i.e., differences in angles between the veins and their relative lengths) and 2) the differences in the amount of laminar outgrowth (for example, the depth of sinuses). Perhaps the reviewer misconstrued the statement to be about the cellular patterning of the vasculature (such as adaxial-abaxial patterning, the developmental

series of xylem maturation, or phloem and companion cell development). I don't think misconstruing is an issue: the context of the paper is so overwhelming about morphometrics that this mistake would not be made by a reader. As of yet, Passiflora is not a developmental genetic system, and only in a few systems (and certainly not Passiflora) has the requisite histology and molecular biology been performed to determine the underlying cellular basis of natural variation in morphology. I would even argue that this remains a grand challenge in leaf developmental genetics overall, because we know so little. Regardless, I do not believe there will be misunderstandings.

p. 3, The authors write that within and between species variation in leaf shape reflects "...both the heteroblastic development of the shoot apical meristem from which they are derived and the ontogeny of individual leaves as they allometrically expand [7-10]." Earlier, in my review of the authors' companion ms, GIGA-D-16-00070, I commented on the authors' repeated references to heteroblasty as resulting "...from the temporal development of the shoot apical meristem, ..." and asked why they emphasized the shoot apical meristem, when (I believe) there is abundant evidence for processes determining shape and venation operating in the developing leaf primordia. Heteroblasty may or may not also reflect progressive changes in the organization of the shoot apical meristem, but whatever the case, a somewhat less facile discussion would be welcome in both mss.

Response: Thank you for your comments. The shoot apical meristem includes both the meristem itself as well as the very earliest leaf primordia. By early leaf primordia, I mean the P0 (plastochron 0, also known as I1, for incipient or invisible leaf 1, in British and continental European works) and ~P1-P2. This definition of shoot apical meristem, which is historically accepted among the plant developmental geneticists, back to Ian Sussex, Wardlaw, the Snows, and earlier, includes both meristem and primordia for the very reasons the reviewer mentions: leaf development is as much about leaf primordia as it is about the meristem (and vice versa). Please see some of the first works on PHANTASTICA by Waites and Hudson, as well as the historical microsurgical works by the authors listed above. It is well accepted that the first patterning events in leaves, including adaxial-abaxial patterning, occur within the P0. The marginal blastozone from which laminar outgrowth occurs, is present in the earliest leaf primordia. The first patterning events that give rise to leaf shape occur in the shoot apical meristem (again, which by definition includes both meristem and the earliest leaf primordia). Please see some of the early works looking at KNOX gene expression and transformations between simple and compound leaves. CUCs, LOBs, RCO, and numerous other factors also regulate leaf dissection in the shoot apical meristem. But this is outside the scope of the manuscript. I could also include the molecular genetics underlying the temporal development of the shoot apical meristem that gives rise to heteroblasty: this includes conserved temporal factors, such as miR156, miR172, their targets SBPs (or SPLs), AP2-likes, and other small RNAs such as the tasiR-ARF pathway. A molecular description of leaf development and heteroblasty is not warranted in this manuscript: it is a mathematical description of leaf shape. In the other companion manuscript, which will now not be published with this manuscript, an extensive description of the historical conception of heteroblasty was provided. This historical background begins with Goethe ("Alles ist Blatt", "Proteus" as the underlying agent of "metamorphosis" in leaves). It continues through Goebel's ideas about the subject, that it might be tied to photosynthesis and sugar. Recently, sugar has been found to be a molecular signal that interacts with miR156/172 to hasten juvenile to adult transitions, and ultimately the reproductive transition. To summarize: there are both historical and modern molecular reasons to describe heteroblasty (which includes traits other than leaf shape too) as the temporal development of the shoot apical meristem from which leaves are derived. Because the second companion manuscript, which will now be published elsewhere, deals mostly with heteroblasty, this discussion will be reserved for that work.

p. 4 (and pp. 12, 13), The authors refer to their analytical results revealing (developmental) constraints on leaf shape and vascular pattern, referring to Fig. 7 and 8. My general reaction to this is to observe that they have achieved a sophisticated description of leaf shape variation in Passiflora, but in my opinion are overly optimistic in suggesting that their results demonstrate process-level constraints. Nevertheless, their example of the close relationships between y- components of landmark 11 and the x-components of landmarks 9 and 13 would support their assertion even better if Fig. 8 could somehow incorporate the sign differences hidden in Fig. 7. It struck me that if lengthwise (y-axis) expansion of the median lobe could be

shown to be associated with contraction of the distal lobes along the x-axis then the authors could well speak of a constraint (conservation of leaf surface area). Such inverse relationships are hard to see, as they require examination of fine
details of Fig. 7, with no help from the vectors in Fig. 8 (no indication whether the correlations coded by the orange color are positive or negative).

Response: This is an excellent comment. I tried to add directionality to the arrows in Fig. 8 as the reviewer mentions. It works well for the example of landmarks 11, 9, and 13 as mentioned (although only one possible direction can be shown). But it did not work well for large groups of correlated landmarks (namely, 1, 2, 3, 4, 5, and 6) only because a single directionality for multiple correlations had to be preserved. The chance that I may make an error depicting these relationships is high. Although not the same thing, the eigen-representations of leaves from the PCA demonstrate the difficulty in depicting these relationships. The hierarchical clustering, of course, remains an accurate depiction of the correlation matrix, but it is too difficult to try to show directionality on the example leaf. But the example leaf still does provide the reader with a depiction of where each landmark is (which is critical).

p. 11, In the Iwata et al. paper (the authors' reference 31) it appeared to me that the observation that the A and D coefficients of the harmonics related to asymmetry was an empirical one. Is there an analytical reason why this should be so? Did the authors carry out the same kind of separate analyses of their EFA data (comparisons of PCAs of the A and D coefficients, and of the B and C coefficients)?

Response: This is an excellent comment. Yes, Iwata et al. does empirically show that B and C harmonics are related to asymmetry, but they also demonstrate why this is theoretically so as well. Because it is mathematical in nature, B and C harmonics strictly represent asymmetry and A and D harmonics strictly symmetry. I have leveraged this theoretical relationship to empirically study asymmetry in references 25, 39, and 40 explicitly and it is an inherent part of EFD analysis regardless. Because all EFD data is always separated into A, B, C, and D harmonics, both symmetry and asymmetry is represented (it’s just that sometimes, by choice, one set of harmonic coefficients is ignored, but not in this study).

pp. 5, 14, The description of the numbering system used ("The numbers written near each leaf..." on p. 14) suggests the reader will have access to the actual scans made by the authors)as their referemnce 45?). Is this in fact the case, and is it necessary? I've suggested above that the authors instead provide vector illustrations of their data (outlines and landmarks), numbered from base to apex of the shoot. The authjors need never confuse their readers with references to how leaves were initially numbered (i.e. in the opposite direction) since that numbering bears no relationship to the heteroblasty that is the subject of the authors' GigaScience mss.

Response: Thank you for this comment. Yes, the readers will have access to the original scans. This is an important part of why this manuscript is going to GigaScience. Ususally, these original scans are not provided, because they are so large, but GigaDB will host them. We are sorry, but originally the scans were numbered from the tip to the base. Because the scans are an integral part of the manuscript, we must make this fact known. Present in the datafiles in GitHub, the total number of nodes per vine collected, plus numbering from the tip to base and base to tip is provided. Of course for analyses in the manuscript, we always use base to tip numbering, but both are provided in the data (and can be converted between each other using the total number of nodes information also provided).
