# Supplementary material for: Morphometric analysis of Passiflora leaves: the relationship between landmarks of the vasculature and elliptical Fourier descriptors of the blade
Source: Gigascience. 2017 Jan 7;6(1):1–13. doi: 10.1093/gigascience/giw008 (PMC5437945; doi:10.1093/gigascience/giw008)
Supplement: Reviewer_1_Report_(Original_Submission).pdf [file giw008_Reviewer_1_Report_(Original_Submission).pdf]

Reviewer Report

Title: "Morphometric analysis of Passiflora leaves: the relationship between landmarks of the vasculature and elliptical Fourier descriptors of the blade"

Version: Original Submission **Date:** 9/15/2016

Reviewer name: Christopher Jiggins

Reviewer Comments to Author:

This paper assesses leaf shape variation in Passiflora using two different morphometric methods. I will say from the start that I am not an expert in these techniques and am not in a position to review the methods or statistics in this paper, so my comments are somewhat superficial and the paper should also be reviewed by a morphometrics/statistical expert.

Background: I'd like to see a bit more justification for the study- Is this aimed at developing automated methods for species identification, or quantifying ecological communities and their diversity of leaf shape? Or is it purely an assessment of the methods for shape quantification (Passiflora have such dramatic shape variation it should be an easy test case?)

The analysis considers 'species' and developmental stage 'heteroblasty' separately and as independent factors. However I think this is the wrong approach because the plasticity of leaves is highly species dependent - so in analysing plasticity, species should be included in the model, such that the interaction of stage and species is considered. It is not really surprising that when considered independently, leaf position is not a very good predictor of leaf shape because the developmental process is different in each species. Or alternatively the leaf position analysis could be conducted on each species separately, if the data are sufficient.

It is interesting that more juvenile leaves show less species-specificity as detected in the dataset - anecdotally I have noticed the same thing.

Discussion: Are there any ecological implications of this work? Are the species sympatric, or where do they come from. I'd be interested to know about disparity in leaf shape among sympatric species, and whether this might be greater than expected by chance, as would perhaps be predicted by the Heliconius coevolution hypothesis. It would be interesting to think further about which ecological questions could be addressed with these methods

Page 3 Line 21 Refer to recent paper on shape learning

Dell'aglio, D. D., Losada, M. E. & Jiggins, C. D. Butterfly Learning and the Diversification of Plant Leaf Shape. Front. Ecol. Evol. 4, 81 (2016).

Level of Interest

Please indicate how interesting you found the manuscript: An article whose findings are important to those with closely related research interests

Quality of Written English

Please indicate the quality of language in the manuscript: Acceptable

Declaration of Competing Interests

Please complete a declaration of competing interests, considering the following questions:

- Have you in the past five years received reimbursements, fees, funding, or salary from an organisation that may in any way gain or lose financially from the publication of this manuscript, either now or in the future?
- Do you hold any stocks or shares in an organisation that may in any way gain or lose financially from the publication of this manuscript, either now or in the future?
- Do you hold or are you currently applying for any patents relating to the content of the manuscript?
- Have you received reimbursements, fees, funding, or salary from an organization that holds or has applied for patents relating to the content of the manuscript?
- Do you have any other financial competing interests?
- Do you have any non-financial competing interests in relation to this paper?

If you can answer no to all of the above, write 'I declare that I have no competing interests' below. If your reply is yes to any, please give details below.

I declare that I have no competing interests

I agree to the open peer review policy of the journal. I understand that my name will be included on my report to the authors and, if the manuscript is accepted for publication, my named report including any attachments I upload will be posted on the website along with the authors' responses. I agree for my report to be made available under an Open Access Creative Commons CC-BY license (http://creativecommons.org/licenses/by/4.0/). I understand that any comments which I do not wish to be included in my named report can be included as confidential comments to the editors, which will not be published.

I agree to the open peer review policy of the journal
